# Complications After Major Surgery for Duodenopancreatic Neuroendocrine Tumors in Patients with MEN1: Results from a Nationwide Cohort

**DOI:** 10.1245/s10434-020-09496-1

**Published:** 2021-01-31

**Authors:** Dirk-Jan van Beek, Sjoerd Nell, Wessel M. C. M. Vorselaars, Bert A. Bonsing, Casper H. J. van Eijck, Harry van Goor, Elisabeth J. Nieveen van Dijkum, Cornelis H. C. Dejong, Gerlof D. Valk, the DutchMEN Study Group (DMSG), P. H. Bisschop, O. M. Dekkers, M. L. Drent, B. Havekes, W. W. de Herder, A. N. A. van der Horst-Schrivers, C. R. C. Pieterman, A. C. van de Ven, Inne H. M. Borel Rinkes, Menno R. Vriens

**Affiliations:** 1 Department of Endocrine Surgical Oncology, University Medical Center Utrecht, Utrecht, The Netherlands; 2 Department of Surgery, Leiden University Medical Center, Leiden, The Netherlands; 3 Department of Surgery, Erasmus Medical Center, Rotterdam, The Netherlands; 4 Department of Surgery, Radboud University Medical Center, Nijmegen, The Netherlands; 5 Department of Surgery, Cancer Center Amsterdam, Amsterdam UMC Academic Medical Center, Amsterdam, The Netherlands; 6 Department of Surgery, Maastricht University Medical Center, NUTRIM School for Nutrition and Translational Research in Metabolism, Maastricht, The Netherlands; 7 Department of Surgery, Universitätsklinikum Aachen, Aachen, Germany; 8 Department of Endocrine Oncology, University Medical Center Utrecht, Utrecht, The Netherlands

## Abstract

**Background:**

Little is known about complications after major duodenopancreatic surgery for duodenopancreatic neuroendocrine tumors (dpNETs) in multiple endocrine neoplasia type 1 (MEN1). Therefore, the incidence and severity of complications after major surgery for MEN1-related dpNETs were assessed.

**Methods:**

Patients were selected from the population-based Dutch MEN1 database if they had undergone a Whipple procedure or total pancreatectomy from 2003 to 2017. Complications were graded according to the Clavien–Dindo classification (grade III or higher complications were considered a severe complication) and definitions from the International Study Group of Pancreatic Surgery. The Cumulative Complication Index (CCI^®^) was calculated as the sum of all complications weighted for their severity. Univariable logistic regression was performed to assess potential associations between predictor candidates and a severe complication.

**Results:**

Twenty-seven patients (median age 43 years) underwent a major duodenopancreatic resection, including 14 Whipple procedures and 13 total pancreatectomies. Morbidity and mortality were 100% (27/27) and 4% (1/27), respectively. A severe complication occurred in 17/27 (63%) patients. The median CCI^®^ was 47.8 [range 8.7–100]. Grade B/C pancreatic fistulas, delayed gastric emptying, bile leakage, hemorrhage, and chyle leakage occurred in 7/14 (50%), 10/27 (37%), 1/27 (4%), 7/27 (26%), 3/27 (11%) patients, respectively. Patients with a severe complication had longer operative time and higher blood loss. After Whipple, new-onset endocrine and exocrine insufficiency occurred in 1/13 and 9/14 patients, respectively.

**Conclusions:**

Major duodenopancreatic surgery in MEN1 is associated with a very high risk of severe complications and cumulative burden of complications and should therefore be reserved for a select subgroup of patients with MEN1-related dpNETs.

**Supplementary Information:**

The online version of this article (10.1245/s10434-020-09496-1) contains supplementary material, which is available to authorized users.

Metastasized duodenopancreatic neuroendocrine tumors (dpNETs) are the leading cause of death in patients with multiple endocrine neoplasia type 1 (MEN1).^1^,^2^ Over 80% of patients are diagnosed with a dpNET by the age of 80 years.^3^,^4^ The majority of pancreatic tumors are clinically silent and are therefore considered non-functioning pancreatic neuroendocrine tumors (NF-pNETs). Duodenal gastrinomas and pancreatic insulinomas are the most frequently encountered hormone-producing dpNETs in MEN1.^5^ Surgical resection is the only potentially curative therapy. Nevertheless, surgery is not recommended for all patients with MEN1-related dpNETs because of the low oncological risk of small NF-pNETs and the equivocal surgical indications for duodenal gastrinomas.^6^^–^9

The decision to proceed to surgery is a risk–benefit balance analysis guided by the oncological benefits against the risks of potential complications and adverse effects. Disease-related factors as well as the young age and postoperative life expectancy of patients with MEN1-related dpNETs influence the timing and extent of surgery. For those patients with duodenal gastrinomas, multifocal dpNETs, or pancreatic head pNETs unsuitable for enucleation, major duodenopancreatic surgery is demanded. A severe complication (Clavien–Dindo grade III or higher) affects one in three patients undergoing pancreatic surgery for MEN1-related NF-pNETs in The Netherlands.^10^ This reflects severe morbidity considering that 80% of the surgical procedures in this cohort included distal pancreatectomies and enucleations.^10^ It is to be expected that the most severe complications occur after more extensive duodenopancreatic resections (i.e. Whipple procedure or total pancreatectomy), resulting in an overall morbidity that is even higher.^10^

Especially if major duodenopancreatic surgery is demanded, the pros and cons should be carefully weighted, but currently clinicians are confronted by a paucity of data regarding postoperative complications and long-term pancreatic function after major duodenopancreatic surgery in MEN1. In addition to the single most severe complication, complications of lesser severity might be clinically relevant and no studies have assessed the cumulative burden of complications in MEN1. Studies on complications after major duodenopancreatic surgery in patients with MEN1 are limited by the low number of pancreatoduodenectomies, reflecting the rarity of the disease, the single-center design, the non-reporting of complications, or the lack of uniform assessment and grading of complications according to currently appraised definitions and grading systems.^10^^–^23 Therefore, this study aimed to assess the incidence, severity, and cumulative burden of postoperative complications and pancreatic function after major duodenopancreatic surgery in a population-based cohort of patients with MEN1. In addition, we aimed to identify potential pre- and intraoperative factors associated with a severe complication.

## Patients and Methods

### Study Design and Patient Selection

Patients were selected from the Dutch MEN1 database, which is owned by the DutchMEN Study Group (DMSG) and has been described in detail before.^24^ Briefly, patients with MEN1 diagnosed according to clinical practice guidelines and aged 16 years and over were included.^6^ Patients were identified in each center based on hospital databases of medical conditions and diseases. More than 90% of MEN1 patients in The Netherlands are included in the database. Clinical and demographic data were collected longitudinally from 1990 to 2017 by standardized medical record review, according to a predefined protocol. The protocol was approved by the Medical Ethics Committees of all University Medical Centers.

Patients undergoing an elective major duodenopancreatic resection from 2003 to 2017 were identified. During the study period, major duodenopancreatic resections were performed in six of eight referral centers in The Netherlands by experienced teams consisting of endocrine and hepato-pancreato-biliary (HPB) surgeons.

A total pancreatectomy is considered a total (duodeno)pancreatic resection. Completion (total) pancreatectomies were defined as Whipple or pylorus-preserving pancreatoduodenectomy (PPPD) after previous distal pancreatectomy or enucleation(s), thus all remaining pancreatic tissue was removed. Whipple/PPPD procedures were performed with or without a distal pancreatectomy. To be classified as Whipple/PPPD plus distal pancreatectomy, preservation of at least a part of the pancreatic body or tail was demanded.

### Clinical Definitions

Patients were operated on for a NF-pNET in case of a pNET on imaging in the absence of excessive hormone production.^8^ Insulinomas were diagnosed based on a 72-h fasting test. Gastrinomas were diagnosed based on hypergastrinemia and a gastrin-positive (duodenal or lymph node) neuroendocrine tumor.^25^ In patients with a gastrinoma and pNET on imaging, the resection was considered for a NF-pNET and gastrinoma.

Data regarding preoperative imaging were collected from conventional imaging, i.e. magnetic resonance imaging (MRI), computed tomography (CT), and endoscopic ultrasonography (EUS). From 2014 onwards, data from gallium-68-labeled imaging were obtained. Tumor size was based on conventional imaging with the shortest time before surgery.^26^ The number of pNETs and presence of lymph node metastases was assessed from both conventional and functional imaging.

Preoperative clinical condition was determined based on the American Society of Anesthesiology (ASA) fitness grade. The duration of surgery was calculated from skin incision until skin closure, and the length of stay (LOS) was computed from the day of surgery until the day of discharge. A readmission was defined as a hospital admission for any surgical complication after discharge. Unplanned intensive care unit (ICU) admission was documented during the initial hospital stay as well as during any readmissions. The number of days on the ICU was calculated from the day of admission until the day of discharge from the ICU. Center volume was defined as high (more than five major resections) or low volume (fewer than five major resections). Period of surgery was stratified into 2003–2010 and 2011–2017.

### Outcomes

The primary outcome of the study was the occurrence of a severe postoperative complication (Clavien–Dindo grade III or higher), since this indicates the need for surgical, radiological, or endoscopic reinterventions.^27^ All complications (i.e. general and pancreatic surgery-specific complications) were graded according to the Clavien–Dindo classification. Morbidity was defined as any complication during the postoperative course (Clavien–Dindo grade I or higher, i.e. any deviation from the normal postoperative course without requiring interventions or pharmacological treatment other than antiemetics, antipyretics, analgesics, diuretics and electrolytes).^27^ Mortality included deaths within 90 days after surgery (Clavien–Dindo grade V).^27^ For every patient, the Comprehensive Complication Index (CCI^®^) score was determined.^28^ The CCI^®^ is calculated as the cumulative sum of all complications weighted for their respective severity and expressed on a continuous scale as a value between 0 (no complication) and 100; patients who die automatically receive a CCI^®^ score of 100.^28^

Secondary outcomes included the presence and severity of pancreatic surgery-specific complications such as postoperative pancreatic fistula (POPF), delayed gastric emptying (DGE), post-pancreatectomy hemorrhage (PPH), bile leak, and chyle leak. These were assessed and graded according to definitions and criteria formulated by the International Study Group of Pancreatic Surgery (ISGPS).^29^^–^33

Patients had pancreatic insufficiency in case of postoperative new-onset diabetes mellitus (endocrine insufficiency) or if patients demanded treatment with pancreatic enzymes (exocrine insufficiency) for at least 6 months. Patients diagnosed with endocrine or exocrine insufficiency preoperatively were excluded from the analysis of new-onset insufficiency. The duration of medication use for pancreatic insufficiency was calculated from the date of the first prescription until the date of withdrawal or date of last follow-up.

### Statistical Analysis

Baseline characteristics are presented as median [range] or counts (percentages). Outcomes are presented for the total cohort and separately for the total pancreatectomy and Whipple/PPPD subgroups. Patients undergoing a total pancreatectomy or completion pancreatectomy were analyzed as total pancreatectomy, and patients undergoing a Whipple/PPPD with or without distal pancreatectomy were studied as Whipple/PPPD. Additionally, the Whipple/PPPD plus distal pancreatectomy subgroup was separately analyzed. Differences in patient, disease, surgical, and intraoperative characteristics were compared between patients with and without a severe complication using Chi square, Fisher’s exact, or Mann–Whitney U tests. Univariable logistic regression was performed to identify factors associated with a severe complication. Variables were selected based on clinical reasoning and included age at surgery (years), sex (female vs. male), center volume (high vs. low), ASA score (1 and 2 vs. 3), tumor size (mm), pNET size ≥ 2 cm (present vs. absent), type of resection (Whipple/PPPD vs. total pancreatectomy), period of surgery (2003–2010 vs. 2011–2017), operative time (minutes), and intraoperative blood loss (mL). Odds ratios (OR) with corresponding 95% confidence intervals (CIs) were calculated. Considering the low number of included patients and low absolute number of patients with a severe complication, multivariable analysis was deemed inappropriate. No missing data were observed for variables assessed by logistic regression. Two-tailed *p*-values < 0.05 were considered statistically significant. Analyses were performed using SPSS version 25.0 (IBM Corporation, Armonk, NY, USA) and R version 3.5.1 (The R Foundation for Statistical Computing, Vienna, Austria).

## Results

### Baseline and Surgical Characteristics

**Fig. 1:**
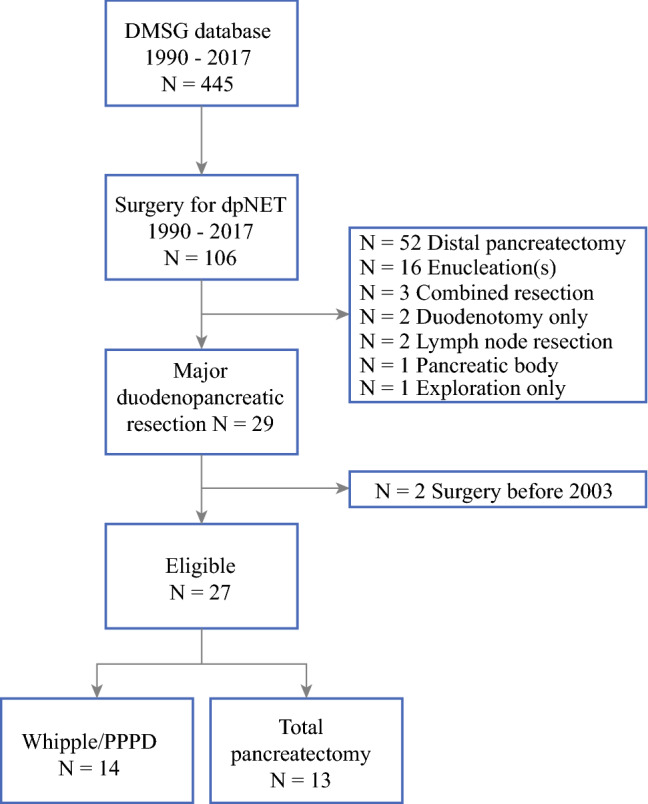
Patient inclusion process. *DMSG* DutchMEN Study Group, *dpNET* duodenopancreatic neuroendocrine tumor, *PPPD* pylorus-preserving pancreatoduodenectomy

A total of 445 patients were identified in the DMSG database, of whom 106 underwent 118 surgical procedures for a dpNET between 1990 and 2018. Twenty-nine patients underwent a major duodenopancreatic resection, of whom two were operated on before 2003 (Fig. 1). Fourteen patients underwent a Whipple/PPPD, and in five of those patients a concurrent distal pancreatectomy was performed, thus leaving a part of the pancreas in situ. Thirteen patients underwent a total pancreatectomy; in four patients, this was considered a completion pancreatectomy after distal pancreatectomy (*n* = 2), enucleation (*n* = 1), or distal pancreatectomy and enucleation (*n* = 1). Two patients underwent a duodenum-preserving total pancreatectomy, and one patient underwent a robot-assisted Whipple/PPPD. Twenty-one patients (78%) were operated within two centers and the remaining six patients were treated in four other hospitals.

Patients underwent surgery at a median age of 43 years [range 28–75] (Table 1). Ten patients (37%) were 40 years or younger at the time of surgery, and the majority of patients (85%; 23/27) had an ASA score of 1 or 2. At the time of surgery, 12 patients (44%) had a suspicion of lymph node metastases, 9 (33%) had a NF-pNET ≥ 2 cm, 3 (11%) had a functioning dpNET, and 3 (11%) had multiple NF-pNETs < 2 cm, respectively (electronic supplementary Table 1). Multiple pNETs on preoperative imaging were observed in 96% (26/27) of patients. The indications for a concomitant distal pancreatectomy were a pNET ≥ 2 cm on preoperative imaging in two patients, an intraoperatively detected pNET of 18 mm, a 15 mm pNET with suspected lymph node metastases on ^68^Gallium-labeled PET/CT, and multiple small pNETs with suspected lymph node metastases.

**Table 1:** Baseline characteristics

Variable	Overall [*n *= 27]	Whipple/PPPD [*n *= 14]	TP [*n *= 13]
Age at surgery, years (median [range])	43.2 [27.5–75.3]	45.4 [29.5–62.5]	42.3 [27.5–75.3]
Sex			
Male	14 (52)	6 (43)	8 (62)
Female	13 (48)	8 (57)	5 (38)
Surgery			
Primary surgery	22 (81)	13 (93)	9 (69)
Reoperation	5 (19)	1 (7)	4 (31)
Surgical indication			
NF-pNET	13 (48)	5 (36)	8 (62)
Insulinoma	2 (7)	0 (0)	2 (15)
Gastrinoma	4 (15)	3 (21)	1 (8)
NF-pNET and gastrinoma	8 (30)	6 (43)	2 (15)
ASA			
1	2 (7)	1 (7)	1 (8)
2	21 (78)	12 (86)	9 (69)
3	4 (15)	1 (7)	3 (23)
Number of pNETs on preoperative imaging (CT, MRI, EUS, PET)			
0	0 (0)	0 (0)	0 (0)
1	1 (4)	1 (8)	0 (0)
2	9 (33)	4 (31)	5 (39)
≥ 3	16 (60)	8 (62)	8 (62)
Size of the largest pNET pancreatic head, mm (median [range])	16.5 [3–42]	20 [3–42]	12 [3–40]
Size of the largest pNET pancreatic body/tail, mm (median [range])	15.5 [3–35]	14.5 [3–35]	18 [5–30]
Suspected lymph node metastases on imaging (CT, MRI, EUS, PET)	12 (44)	9 (64)	3 (23)
Type of resection			
Whipple/PPPD	14 (52)	NA	NA
Whipple/PPPD plus distal pancreatectomy^a^	5 (36)		
Total pancreatectomy	13 (48)		
Completion pancreatectomy	5 (38)		
Period of surgery			
2003–2010	9 (33)	4 (29)	5 (38)
2011–2017	18 (66)	10 (71)	8 (62)
Lymph node resection	22 (81)	13 (93)	9 (69)
Approach			
Conventional	26 (96)	13 (93)	13 (100)
Robot-assisted	1 (4)	1 (7)	0 (0)

Data are expressed as *n* (%) unless otherwise specified

*ASA* American Society of Anesthesiology, *CT* computed tomography, *EUS* endoscopic ultrasonography, *MRI* magnetic resonance imaging, *NA* not applicable, *NF*-*pNET* non-functioning pancreatic neuroendocrine tumor, *PET* positron emission tomography, *pNET* pancreatic neuroendocrine tumor, *PPPD* pylorus-preserving pancreatoduodenectomy, *TP* total pancreatectomy

^a^In order to be classified as Whipple/PPPD plus distal pancreatectomy, a part of the pancreatic body or tail had to be left in situ

More procedures were performed from 2011 to 2017 than from 2003 to 2010 (18/27 [67%] vs. 9/27 [33%]). Seventy-one percent (10/14) of the Whipple/PPPD procedures and 62% (8/13) of the total pancreatectomies, of which four were completion pancreatectomies, were performed from 2011 to 2017.

### Occurrence of Complications

Intraoperative outcomes and characteristics of hospital stay are shown in Table 2. Morbidity and mortality were 100% (27/27) and 4% (1/27), respectively (Table 3). The only death occurred in a patient 30 days after total pancreatectomy. Although complicated by ascites, thrombocytosis, and a magnesium calcium electrolyte disorder, the exact cause of death remained unknown after autopsy.

**Table 2:** Intraoperative characteristics and hospital stay

	Overall [*n* = 27]	Whipple/PPPD [*n* = 14]	Total pancreatectomy [*n* = 13]
Time of surgery, min (median [range])	304 [183–480]	315 [198–425]	304 [183–480]
Blood loss, mL (median [range])	825 [100–3350]	500 [100–2000]	900 [200–3350]
Length of stay, days (median [range])	16 [7–291]	16.5 [7–78]	16 [10–291]
Re-laparotomy [*n* (%)]	3 (11)	1 (7)	2 (15)
ICU admission [*n* (%)]	7 (26)	3 (21)	4 (31)
Duration of ICU admission, days (median [range])	1 [1–46]	8 [1–46]	1 [1–6]
Readmission [*n* (%)]	10 (37)	4 (29)	6 (46)
Duration of readmission, days (median [range])	12.5 [2–72]	18 [2–31]	12.5 [5–72]
ICU during readmission [*n* (%)]	5 (19)	2 (14)	3 (23)
ICU during readmission duration, days (median [range])	4 [2–69]	2 [2]	10 [4–69]

*ICU* intensive care unit, *PPPD* pylorus-preserving pancreatoduodenectomy

**Table 3:** Severity of postoperative complications according to the Clavien–Dindo classification

Outcomes	Overall [*n *= 27]	Whipple/PPPD [*n *= 14]			Total pancreatectomy [*n *= 13]
		Overall [*n *= 14]	Whipple/PPPD only [*n *= 9]	Whipple/PPPD plus distal pancreatectomy [*n *= 5]	
Clavien–Dindo grade					
I	4 (15)	2 (14)	2 (22)	0 (0)	2 (15)
II	6 (22)	1 (7)	1 (11)	0 (0)	5 (29)
IIIA	6 (22)	5 (36)	2 (22)	3 (60)	1 (8)
IIIB	1 (4)	1 (7)	0 (0)	1 (20)	0 (0)
IVA	7 (26)	4 (29)	3 (33)	1 (20)	3 (23)
IVB	2 (7)	1 (7)	1 (11)	0 (0)	1 (8)
V	1 (4)	0 (0)	0 (0)	0 (0)	1 (8)
Clavien–Dindo grade III or higher complications	17 (63)	11 (79)	6 (67)	5 (100)	6 (46)
Multiple Clavien–Dindo grade III or higher complications	13 (48)	9 (64)	4 (44)	5 (100)	4 (31)
Clavien–Dindo grade III	7 (26)	6 (43)	2 (22)	4 (80)	1 (8)
Clavien–Dindo grade III specified	POPF (1)	POPF (1)	DGE (1)		
	POPF + anastomotic leakage (1)	POPF + anastomotic leakage (1)			
	POPF + DGE (1)	POPF + DGE (1)			
	POPF + bile leakage (1)	POPF + bile leakage (1)			
	POPF + PPH + abdominal abscess + wound infection/abscess (1)	POPF + PPH + abdominal abscess + wound infection/abscess (1)			
	Chyle leakage (1)	DGE (1)			
	DGE (1)				
Clavien–Dindo grade IV	9 (33)	5 (36)	4 (44)	1 (20)	4 (31)
Clavien–Dindo grade IV specified	PPH (4) Respiratory insufficiency (2) Cardiac tamponade (1) Esophageal perforation + PPH (1) POPF + PPH (1)	PPH (2) Respiratory insufficiency (2) POPF + PPH (1)			PPH (2) Cardiac tamponade (1) Esophageal perforation + PPH (1)
Clavien–Dindo grade V	1 (4)	0 (0)	0 (0)	0 (0)	1 (8)
CCI (median [range])	47.8 [8.7–100]	50.6 [8.7–95.3]	35.7 [8.7–95.3]	54.8 [47.8–69.2]	32.0 [12.2–100]
CCI					
0–19	5 (19)	2 (14)	2 (22)	0 (0)	3 (23)
20–39	7 (26)	3 (21)	3 (33)	0 (0)	4 (31)
40–59	7 (26)	5 (36)	1 (11)	4 (80)	2 (15)
60–79	5 (19)	3 (21)	2 (22)	1 (20)	2 (15)
≥ 80	3 (11)	1 (7)	1 (11)	0 (0)	2 (15)

Data are expressed as *n* (%) unless otherwise specified

*CCI* Cumulative Complication Index, *DGE* delayed gastric emptying, *POPF* postoperative pancreatic fistula, *PPH* post-pancreatectomy hemorrhage, *PPPD* pylorus-preserving pancreatoduodenectomy

A severe complication (Clavien–Dindo grade III or higher) occurred in 17 patients (63%), of whom 13 (76%) developed at least one more severe complication. A severe complication occurred in 6/9 (67%) patients after a Whipple/PPPD alone, 5/5 (100%) patients after a Whipple/PPPD plus distal pancreatectomy, 4/8 (50%) patients after a total pancreatectomy, and 2/5 (40%) patients after a completion pancreatectomy. All patients who underwent a Whipple/PPPD plus distal pancreatectomy developed multiple severe complications (Table 3). Median hospital stay was 19 days [range 10–291] for patients who developed a severe complication and 14.5 days [range 8–30] for patients without a severe complication.

**Fig. 2:**
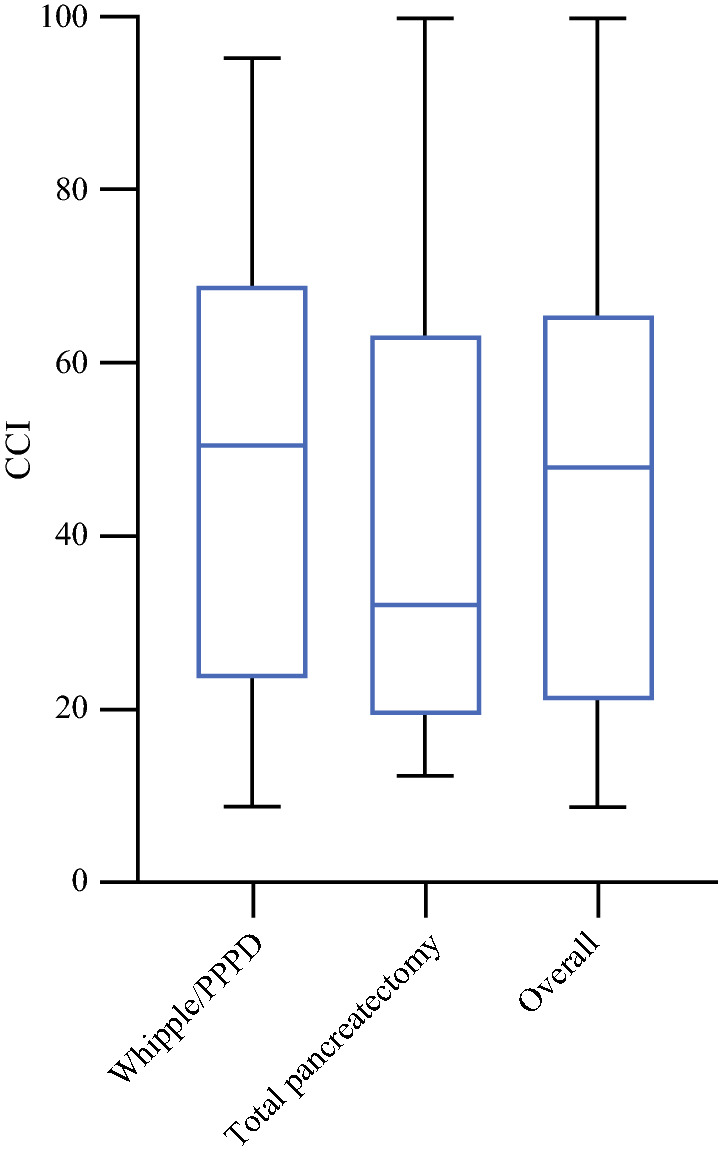
Boxplots of the CCI^®^ for the total cohort and stratified by procedure. *CCI*^*®*^ Cumulative Complication Index, *PPPD* pylorus-preserving pancreatoduodenectomy

The CCI^®^ is presented in Fig. 2 and Table 3. The overall median CCI^®^ was 47.8 [range 8.7–100] and the median CCI^®^ was higher for patients in the Whipple/PPPD group (50.6 [range 8.7–95.3]) compared with patients in the total pancreatectomy group (32.0 [range 12.2–100]). A CCI^®^ ≥ 50 was observed in seven patients (50%) after a Whipple/PPPD and five (38%) patients after a total pancreatectomy. The CCI^®^ of individual patients is visualized in electronic supplementary Fig. 1.

### Occurrence of Pancreatic Surgery-Specific Complications According to the International Study Group of Pancreatic Surgery Definitions and Grading

Pancreatic surgery-specific complications according to the ISGPS definitions and grading are presented in Table 4. Half of the patients (7/14) undergoing a Whipple/PPPD developed a POPF, of whom 6/7 (86%) were grade B. In terms of percentages, more patients in the Whipple/PPPD group had DGE grade C compared with the total pancreatectomy group (5/14 [36%] vs. 1/13 [8%]). Grade B/C PPH occurred in 26% (7/27) of patients. Three patients underwent re-laparotomy for PPH—one underwent re-laparotomy for a splenic artery bleeding in an emergency setting (hypovolemic shock and resuscitation), one had a resection of the jejunum for severely bleeding ulcers, and one had evacuation of multiple hematomas. The source of PPH could be adequately coiled in three patients and one patient was managed conservatively. Within the patients who underwent a Whipple/PPPD plus distal pancreatectomy, POPF, DGE, bile leakage, PPH, and chyle leakage occurred in 4/5 (80%), 5/5 (100%), 1/5 (20%), 2/5 (40%) and 0/5 (0%) patients (Table 4).

**Table 4:** Pancreatic surgery-associated complications according to the ISGPS definitions and grading

Complication	Overall [*n *= 27]	Whipple/PPPD [*n *= 14]			Total pancreatectomy [*n *= 13]
		Overall [*n *= 14]	Whipple/PPPD only [*n *= 9]	Whipple/PPPD plus distal pancreatectomy [*n *= 5]	
POPF	7 (26)	7 (50)	3 (33)	4 (80)	NA
POPF grade					
B	6 (22)	6 (86)	2 (67)	4 (100)	NA
C	1 (4)	1 (14)	1 (33)	0 (0)	
DGE	19 (70)	10 (71)	5 (56)	5 (100)	9 (69)
DGE grade					
A	9 (33)	4 (40)	2 (40)	2 (40)	5 (56)
B	4 (15)	1 (10)	0	1 (20)	3 (33)
C	6 (22)	5 (50)	3 (60)	2 (40)	1 (11)
Bile leakage	4 (15)	2 (14)	1 (11)	1 (20)	2 (15)
Bile leakage grade					
A	3 (11)	1 (50)	1 (100)	0 (0)	2 (100)
B	1 (4)	1 (50)	0 (0)	1 (100)	0 (0)
C	0 (0)	0 (0)	0 (0)	0 (0)	0 (0)
PPH	7 (26)	4 (29)	2 (22)	2 (40)	3 (23)
PPH grade					
A	0 (0)	0 (0)	0	0	0 (0)
B	3 (11)	1 (25)	0	1 (50)	2 (67)
C	4 (15)	3 (75)	2 (100)	1 (50)	1 (33)
Chyle leakage	4 (15)	3 (21)	3 (33)	0	1 (8)
Chyle leakage grade					
A	1 (4)	1 (33)	1 (33)	0	0 (0)
B	3 (11)	2 (66)	2 (67)	0	1 (100)
C	0 (0)	0 (0)	0	0	0 (0)

Data are expressed as *n* (%)

*DGE* delayed gastric emptying, *ISGPS* International Study Group of Pancreatic Surgery, *NA* not applicable, *POPF* postoperative pancreatic fistula, *PPH* post-pancreatectomy hemorrhage, *PPPD* pylorus-preserving pancreatoduodenectomy

### Factors Associated with a Severe Complication

Regarding preoperative characteristics, a severe complication was more frequently observed in males compared with females (65% vs. 35%, *p* = 0.09) and after a Whipple/PPPD compared with a total pancreatectomy (65% vs. 35%, *p* = 0.09) [electronic supplementary Table 2]. No meaningful differences were observed for age at surgery, tumor size, the presence of a pNET ≥ 2 cm, ASA score, center volume, and period of surgery (electronic supplementary Table 2). Patients with a severe complication had a longer operative time (356 vs. 265.5 min, *p* = 0.01) [OR 1.19, 95% CI 1.05–1.41] per 10 min, and intraoperative blood loss was higher in patients developing a severe complication (900 vs. 425 mL, *p* = 0.02) [OR 1.22, 95% CI 1.04–1.59] per 100 mL.

### Long-Term Pancreatic Function

The exocrine and endocrine pancreatic insufficiency frequencies are reported in Table 5. Of the patients undergoing a Whipple/PPPD with and without concurrent distal pancreatectomy, 67% (6/9) and 60% (3/5) had pancreatic insufficiency postoperatively, respectively. After a Whipple/PPPD, 1/13 patients (8%; one patient suffered from diabetes preoperatively) developed new-onset diabetes without exocrine insufficiency, whereas exocrine insufficiency after Whipple/PPPD occurred in 9/14 (64%) patients. Overall, patients were taking insulin for a median of 5.5 years [range 0–13.8] and taking pancreatic enzymes for a median of 5.1 years [range 0–13.8]. After a Whipple/PPPD, none of the patients could stop their endocrine or exocrine treatment during follow-up.

**Table 5:** Occurrence of pancreatic exocrine and endocrine insufficiency stratified by type of resection

Resection	No insufficiency	Endocrine only	Exocrine only	Both
Whipple/PPPD [*n* = 14]	5 (36)	0 (0)	7 (50)	2 (14)^b^
Whipple/PPPD only [*n* = 9]	3 (33)	0 (0)	5 (56)	1 (11)
Whipple/PPPD plus distal pancreatectomy [*n* = 5]^a^	2 (40)	0 (0)	2 (40)	1 (20)^b^
Total pancreatectomy [*n* = 13]	0 (0)	0 (0)	0 (0)	13 (100)^c^

Data are expressed as *n* (%)

*PPPD* pylorus-preserving pancreatoduodenectomy

^a^In order to be classified as Whipple/PPPD plus distal pancreatectomy, a part of the pancreatic body or tail had to be preserved in situ

^b^One patient already had diabetes before surgery

^c^Two patients had diabetes before total pancreatectomy/completion pancreatectomy

## Discussion

Within this nationwide cohort of patients undergoing major duodenopancreatic surgery for MEN1-related dpNETs, rates of morbidity, severe morbidity, and mortality were 100%, 63%, and 4%, respectively. These results demonstrate that, even in high-volume academic HPB centers, major duodenopancreatic surgery in patients with MEN1 is associated with a very high rate of severe complications and cumulative burden of morbidity, and underscore the importance of patient selection and adequate preoperative patient counseling. No preoperative patient characteristics were associated with complications. If patients are exposed to major duodenopancreatic surgery, longer duration of surgery and more blood loss warrant more intensified perioperative care.

Previous studies investigating complications after pancreatic surgery for MEN1-related dpNETs observed complication rates ranging from 26 to 58%;^11^,^13^^–^17^,^^23^^,^^34^ however, complications were not systematically addressed according to accepted classification systems in the majority of studies. The only study that has overcome these issues was previously conducted by our group and observed a relatively high rate of severe complications of 33%. This high rate can be explained by the fact that complications were the primary outcome of this study and because all complications in individual patients were systematically scored.^10^ Few other studies have described complications after MEN1-associated major duodenopancreatic surgery. Lopez et al. included 13 patients, of whom 4 (31%) developed a complication;^11^ Bartsch et al. observed complications in 3 of 4 (75%) patients;^17^ Vezzosi et al.^14^ included 9 patients, of whom 6 (67%) developed a short-term complication; and Tonelli et al. investigated 14 patients, of whom 5 (36%) developed an abdominal complication.^16^ These low numbers of patients express the rarity of the disease. In comparison with the previous DMSG study on complications after pancreatic surgery in the setting of MEN1, the currently observed percentage of 63% of severe complications most likely reflects the high percentage of major pancreatoduodenectomies, and indicates that this specific subgroup carries a formidable risk of developing severe complications. This is also underscored by the high median CCI^®^ of 47.8 [range 8.7–100], compared with a median CCI^®^ of 20.9 [range 0–33.5] in benchmark cases, defined as patients without significant comorbidities and major vascular resection, across 23 high-volume centers for pancreatic surgery.^35^

Although current MEN1 guidelines do not routinely recommend Whipple procedures because of an increased operative mortality and long-term morbidity, these recommendations are not substantiated by underlying scientific evidence in patients with MEN1.^6^ A systematic review investigating POPF after Whipple/PPPD in non-MEN1 patients, observed POPF grade B/C in 22–26% of patients.^36^ Patients with MEN1 have multiple risk factors, i.e. soft pancreas, pathology (neuroendocrine tumor), small pancreatic duct, for POPF and combined procedures are often performed due to the multiplicity of dpNETs.^37^^,^^38^ Three of the nine Whipple patients and four of five patients undergoing a Whipple/PPPD plus distal pancreatectomy developed a POPF grade B/C. On the contrary, approximately one in three patients (36%) in the Whipple/PPPD group did not suffer from pancreatic insufficiency, and the incidence was similar between the Whipple/PPPD only and Whipple/PPPD plus distal pancreatectomy groups. A recent study suggested that the risk of exocrine pancreatic insufficiency is related to the type of procedure and not the underlying hereditary syndrome.^39^ Although no comparison between underlying pNET etiology was performed in the present study, the percentages of exocrine insufficiency were relatively similar between both studies—six of nine patients in the Whipple/PPPD-only group in this study, compared with three of six patients in the cohort described by McDonald et al.^39^ Future studies should evaluate the differences between patients with MEN1-related and sporadic pNETs, also adjusting for age and the estimated volume of the remnant pancreas.

At present, MEN1 clinical practice, European Neuroendocrine Tumor Society (ENETS), and North American Neuroendocrine Tumor Society (NANETS) guidelines recommend surgery for MEN1-related functioning pNETs.^6^,^40^,^41^ For NF-pNETs larger than 2 cm, surgery is advised by the guidelines, which is also substantiated by population-based cohort studies in MEN1.^8^,^9^,^40^^–^43 Medical management is indicated in most patients with gastrinomas; surgical indications include failure of medical therapy, a pancreatic gastrinoma, a pNET larger than 2 cm, and lymph node metastases.^6^,^40^^,^^41^ Due to the increased use of ^68^Gallium-labeled PET/CT, more lymph node metastases might be detected, thus increasing the number of patients fulfilling the criteria for (extensive) surgery.^44^ Within this series, the majority of patients had suspected lymph node metastases preoperatively, or single or multiple pNETs ≥ 2 cm, indicating that most patients had a surgical indication according to the current insights.

Besides surgical indications, the ENETS guideline recommends enucleations or limited resections whenever possible and MEN1 guidelines discourage the routine use of Whipple procedures.^6^^,^^40^ Although enucleations or limited resections provide a cure for MEN1-related pNETs, in some patients a Whipple/PPPD seems appropriate.^15^^,^^34^^,^^45^ Arguments in favor of a Whipple/PPPD instead of an enucleation of the pancreatic head include the required technical feasibility of an enucleation (i.e. > 2–3 mm from the main pancreatic duct), multiple pNETs in the head, concurrent (duodenal) gastrinomas, as well as suspected lymph node metastases. Patients with diffuse pNETs throughout the pancreas pose a specific challenge for surgeons, especially if multiple pNETs fulfill the criteria for operative resection. Instead of a total pancreatectomy or Whipple plus distal pancreatectomy, an enucleation plus distal pancreatectomy could be considered to reduce the risk of early and late complications. Based on currently accepted prognostic factors, a concomitant distal pancreatectomy can be considered based on the size, growth rate, or tumor grade, in case of a functioning pNET or if lymph nodes are suspected to originate from a pancreatic tail tumor. However, in these situations, the risk of malignancy of every individual dpNET is preferably estimated, but the major unmet need for MEN1-related NF-pNETs and gastrinomas is proper risk stratification.^46^ Recent insights into the presence of alpha and beta cell subtypes—based on transcription factors ARX and PDX1—of MEN1-related pNETs should be used for risk stratification and patient selection for major surgery.^47^ Prognostic factors such as World Health Organization (WHO) grade, alternative lengthening of telomeres, DNA methylation, and expression of p27^Kip1^ and p18^Ink4c^ can additionally be taken into account.^47^^–^50 Molecular prognostic factors for gastrinomas have been far less developed, and therefore patient selection should be guided by more traditional and readily available factors, such as gastrin levels, (aggressive) tumor growth, and pNET size.^1^,^9^,^20^,^25^,^51^,^52^ The high risk of severe complications underscores the need for better risk stratification for MEN1-related dpNETs, therefore major surgery will be offered selectively in the future.

Although all of the procedures were performed in tertiary referral centers by experienced surgeons with a vast experience in endocrine and HPB surgery, some centers performed only one to three procedures, whereas 78% were performed in two centers. Hence, further centralization of these rare and extremely complex cases within the landscape of endocrine and HPB surgery should be encouraged.^53^ Nationwide centralization of pancreatic surgery for adenocarcinomas has decreased the rates of postoperative complications in The Netherlands.^54^ In addition, preoperative patient selection is of utmost importance to expose only those who will clinically benefit. Preoperative assessment by multidisciplinary teams with vast experience in both neuroendocrine tumors and MEN1 could aid in selecting the right patients for these high-risk procedures.^55^ Distant metastases, which reduce the prospects of curative surgery, are ideally excluded preoperatively on ^68^Gallium-labeled PET/CT, particularly when major surgery is considered. Considering the very high risk of severe complications, referral to experienced endocrine surgeons to explore the technical possibilities of limited resections without compromising oncologic outcomes is of utmost importance, especially since no risk factors were observed to preoperatively identify patients with post-surgery severe complications. If patients undergo major surgery, well-known intraoperative factors (blood loss and operative time) might identify patients with a higher risk of a severe complication, and subsequently warrant close perioperative monitoring to enable early identification and timely treatment of complications.

A recent study reported lanreotide to be more effective than active surveillance for MEN1-related pNETs < 2 cm;^56^ however, patients were free to choose either lanreotide or active surveillance, and thus baseline characteristics differed between both groups. In addition, the rate of progression in the lanreotide group was similar to that observed in population-based cohorts from France and The Netherlands without treatment.^8^^,^^43^ Although the major unmet need is adequate risk stratification and patient selection for surgery, alternative treatment options can be considered in individual patients unwilling or unfit to undergo major surgery. Nevertheless, these therapies, such as lanreotide, should be investigated in randomized controlled trials.

The strengths of this study include the population-based cohort, the relatively high number of patients in spite of the rarity of disease, and the systematic assessment and grading of postoperative complications according to generally accepted classifications and definitions in each individual patient. In addition, this study provides insight into the cumulative burden of complications in MEN1. Furthermore, due to the nationwide collaboration, loss to follow-up is prevented, indicating that data were available even if patients were readmitted for complications to other university hospitals. Limitations include the high number of centers and subsequent number of surgeons who performed the procedures. Although 12 patients were previously described, by expanding indications to all dpNETs and extending the inclusion period up to 2018, the number of eligible patients could more than double.^10^ Furthermore, the relatively long time span of 15 years for patient inclusion is prone to changes in patient care. This is also reflected in the surgical indications, which showed an increase in Whipple procedures and a decrease in total pancreatectomies (excluding completion pancreatectomies) in the most recent period. Although this study aimed to investigate the incidence and severity of postoperative complications after major duodenopancreatic surgery in MEN1, a direct comparison of different pancreatic procedures, taking a broad spectrum of early complications as well as long-term outcomes, such as survival, liver metastases, and the occurrence of new dpNETs, would be interesting since this might further contribute to evidence-based surgical decision making regarding the extent of surgery.

## Conclusion

Major duodenopancreatic surgery is associated with a high rate of severe complications and cumulative morbidity and should therefore be reserved for a select subgroup of patients with MEN1-related dpNETs. These data underscore the need for adequate risk stratification for MEN1-related dpNETs. Patients should be discussed in multidisciplinary tumor boards with vast experience in MEN1-related dpNETs. In addition, individual surgical decision making should be undertaken in conjunction with the patients and their families, carefully weighing the pros and cons of major duodenopancreatic surgery.

## Electronic supplementary material

Below is the link to the electronic supplementary material.

Supplementary material 1 (DOCX 34 kb)

Supplementary Fig. 1 CCI^®^ per individual patient. CCI^®^ Cumulative Complication Index (TIFF 1021 kb)

## Data Availability

Because of the sensitive nature of the data collected for this study, the authors do not wish to make the data publicly available.
