# Supplementary material for: Complications After Major Surgery for Duodenopancreatic Neuroendocrine Tumors in Patients with MEN1: Results from a Nationwide Cohort
Source: Ann Surg Oncol. 2021 Jan 31;28(8):4387–99. doi: 10.1245/s10434-020-09496-1 (PMC8253708; doi:10.1245/s10434-020-09496-1)
Supplement: Supplementary file 1 — Supplementary material 1 (DOCX 34 kb) [file 10434_2020_9496_MOESM1_ESM.docx]

| **SUPPLEMENTARY TABLE 1** Surgical indications and preoperative imaging | | | | | | |
| --- | --- | --- | --- | --- | --- | --- |
| # | Surgery | Indication | Gastrinoma^a^ | pNET Head Size (mm) | pNET Body/ Tail Size (mm) | LNM |
| 1 | Whipple/PPPD | Gastrinoma | + | 10 | *13* | – |
| 2 | Whipple/PPPD | NF-pNET |  | 22 |  | – |
| 3 | Whipple/PPPD | Gastrinoma |  | 8 | *18* | + |
| 4 | Whipple/PPPD | NF-pNET |  | 26 | *13* | – |
| 5 | Whipple/PPPD | NF-pNET + Gastrinoma | + | 18 |  | + |
| 6 | Whipple/PPPD | NF-pNET |  | 23 | 35^b^ | + |
| 7 | Whipple/PPPD | NF-pNET |  |  | *10* | + |
| 8 | Whipple/PPPD | NF-pNET + Gastrinoma | + | 10 | *14* | + |
| 9 | Whipple/PPPD | Gastrinoma | + | 8 |  | + |
| 10 | Whipple/PPPD + DP | NF-pNET + Gastrinoma |  | 29 | 20 | + |
| 11 | Whipple/PPPD + DP | NF-pNET + Gastrinoma |  | 3 | 3 | – |
| 12 | Whipple/PPPD + DP | NF-pNET |  | 34 |  | – |
| 13 | Whipple/PPPD + DP | NF-pNET + Gastrinoma | + | 20 | 20 | + |
| 14 | Whipple/PPPD + DP | NF-pNET + Gastrinoma | + | 42 | 15 | + |
| 15 | TP | NF-pNET |  |  | 22 | – |
| 16 | TP | Insulinoma |  | 20 | 21 | – |
| 17 | TP | NF-pNET + Gastrinoma |  | 5 | 9 | – |
| 18 | TP | NF-pNET |  | 12 | 5 | – |
| 19 | TP | NF-pNET | – | 15 | 20 | – |
| 20 | TP | Insulinoma |  | 25 | 16 | – |
| 21 | TP | NF-pNET |  | 9 | 30 | – |
| 22 | TP | NF-pNET + Gastrinoma | + |  | >20 | + |
| 23 | TP/CP | Gastrinoma | – | 3 | 7 | + |
| 24 | TP/CP | NF-pNET |  | 11 | *NA* | + |
| 25 | TP/CP | NF-pNET |  | 8 | 21 | – |
| 26 | TP/CP | NF-pNET | – | 40 | *NA* | – |
| 27 | TP/CP | NF-pNET |  | 20 | 10 | – |
| Data given based on preoperative radiology  ^a^ Based on Gallium-68-labeled PET/CT  ^b^ This patient underwent an extended Whipple/PPPD with resection of the 35mm pancreatic body tumor  *CP* completion pancreatectomy, *DP* distal pancreatectomy, *F* female, *LNM* lymph nodes metastasis, *M* male, *mm* millimeter, *NF-pNET* non-functioning pancreatic neuroendocrine tumor, *pNET* pancreatic neuroendocrine tumor, *PPPD* pylorus-preserving pancreatoduodenectomy, *TP* total pancreatectomy | | | | | | |

| **SUPPLEMENTARY TABLE 2** Factors associated with a severe complication | | | | | |
| --- | --- | --- | --- | --- | --- |
| Variable | Severe complication  [*n* = 17] | No severe complication  [*n* = 10] | *p*-Value | Odds ratio | 95% CI |
| Age at surgery, years | 43.2 [29.5–61.8] | 46.4 [27.5–75.3] | 0.54 | 0.97 | 0.91–1.04 |
| Sex  Female  Male | 6 (35%)  11 (65%) | 7 (70%)  3 (30%) | –  0.09 | 1.00 (Ref.)  4.28 | Ref.  0.85–26.3 |
| Center volume  High  Low | 13 (76%)  4 (24%) | 8 (80%)  2 (20%) | –  0.83 | 1.00 (Ref.)  1.23 | Ref.  0.19–10.38 |
| ASA score  1  2  3 | 1 (6%)  13 (77%)  3 (18%) | 1 (10%)  8 (80%)  1 (10%) | –  0.74  0.55 | 1.00 (Ref.)  1.63  3.00 | Ref.  0.06–45.14  0.07–170.8 |
| ASA score  1–2  3 | 4 (82%)  3 (18%) | 9 (90%)  1 (10%) | –  0.59 | 1.00 (Ref.)  1.93 | Ref.  0.21–42.42 |
| Size of the largest pNET, mm | 20 [3–42] | 20.5 [7.2–40] | 0.86 | 1.01 | 0.93–1.09 |
| pNET ≥2 cmr  No  Yes | 7 (41%)  10 (59%) | 4 (40%)  6 (60%) | –  0.95 | 1.00 (Ref.)  0.95 | Ref.  0.18–4.69 |
| Type of resection  Total pancreatectomy Whipple/PPPD | 6 (35%)  11 (65%) | 7 (70%)  3 (30%) | –  0.09 | 1.00 (Ref.)  4.28 | Ref.  0.85–26.3 |
| Period of surgery  2003–2010  2011–2017 | 4 (24%)  13 (77%) | 5 (50%)  5 (50%) | –  0.17 | 1.00 (Ref.)  3.25 | Ref.  0.62–18.72 |
| Time of surgery, min | 356 [204–650] | 265.5 [183–390] | 0.01 | 1.19^a^ | 1.05–1.41 |
| Intraoperative blood loss, mL | 900 [250–3350] | 425 [100–1400] | 0.02 | 1.22^b^ | 1.04–1.59 |
| ^a^ Per 10 min  ^b^ Per 100 mL  All continuous variables are presented as median [range]  *ASA* American Society of Anesthesiology, *CI* confidence interval, *pNET* pancreatic neuroendocrine tumor, *PPPD* pylorus-preserving pancreatoduodenectomy | | | | | |
