# Supplementary figures and images for: Complications After Major Surgery for Duodenopancreatic Neuroendocrine Tumors in Patients with MEN1: Results from a Nationwide Cohort
Source: Ann Surg Oncol. 2021 Jan 31;28(8):4387–99. doi: 10.1245/s10434-020-09496-1 (PMC8253708; doi:10.1245/s10434-020-09496-1)

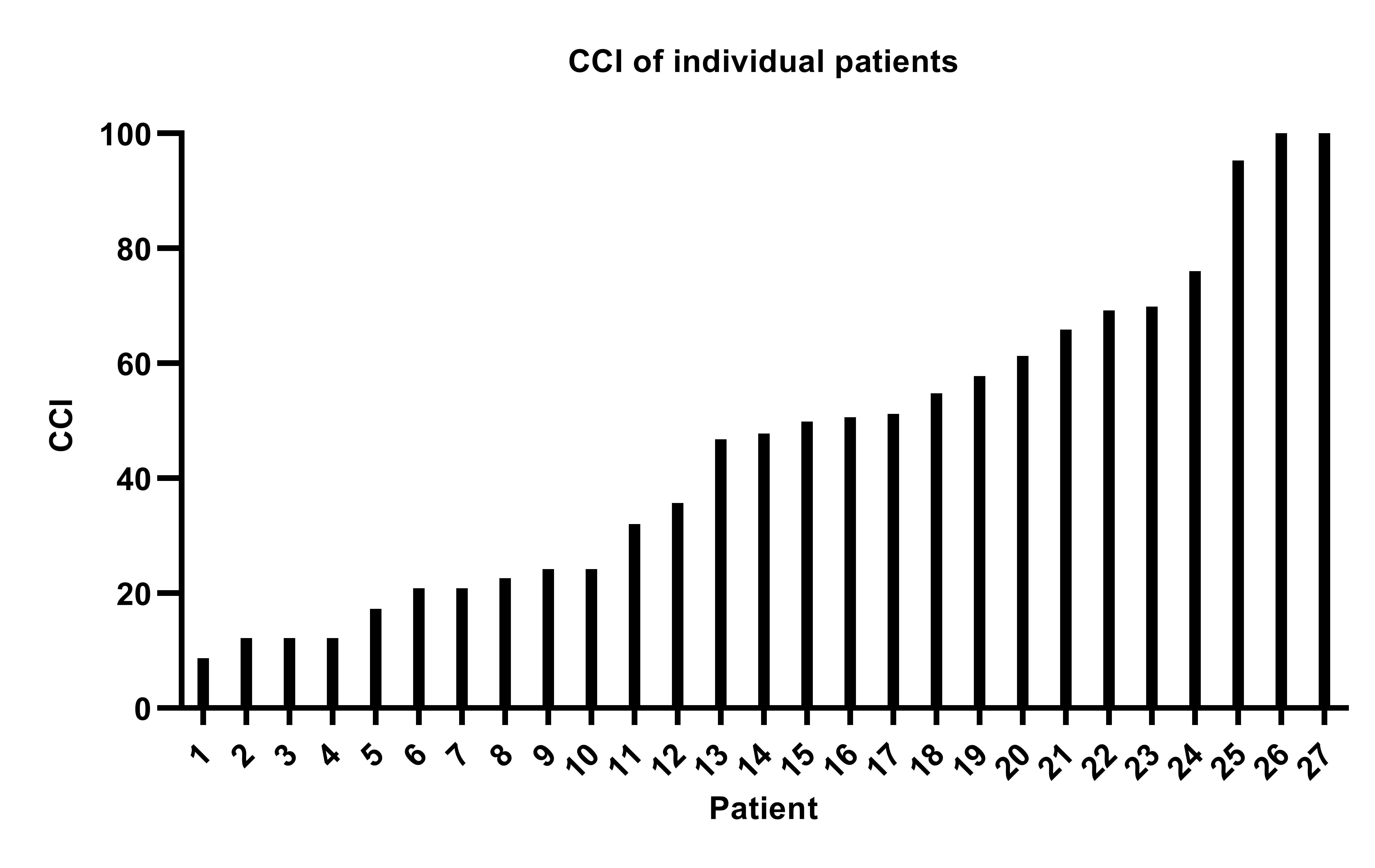

Supplement: Supplementary file 2 — CCI® per individual patient. CCI® Cumulative Complication Index (TIFF 1021 kb) [file 10434_2020_9496_MOESM2_ESM.tif]
